# Sex work and the 2010 FIFA World Cup: time for public health imperatives to prevail

**DOI:** 10.1186/1744-8603-6-1

**Published:** 2010-02-11

**Authors:** Marlise L Richter, Matthew F Chersich, Fiona Scorgie, Stanley Luchters, Marleen Temmerman, Richard Steen

**Affiliations:** 1 International Centre for Reproductive Health, Department of Obstetrics and Gynaecology, Ghent University, De Pintelaan 185, Ghent 9000, Belgium; 2 Forced Migration Studies Programme, University of the Witwatersrand, 1 Jan Smuts Avenue, Johannesburg, 2000, South Africa; 3 Centre for Health Policy, School of Public Health, University of the Witwatersrand, No 7 York Avenue, Parktown, Johannesburg, 2193, South Africa; 4 Independent consultant, PO Box 568, Cramerview, Johannesburg, 2060, South Africa; 5 Independent consultant, 3 Blenheim Mansions, Brixton Hill, London SW2 1SA, UK

## Abstract

**Background:**

Sex work is receiving increased attention in southern Africa. In the context of South Africa's intense preparation for hosting the 2010 FIFA World Cup, anxiety over HIV transmission in the context of sex work has sparked debate on the most appropriate legal response to this industry.

**Discussion:**

Drawing on existing literature, the authors highlight the increased vulnerability of sex workers in the context of the HIV pandemic in southern Africa. They argue that laws that criminalise sex work not only compound sex workers' individual risk for HIV, but also compromise broader public health goals. International sporting events are thought to increase demand for paid sex and, particularly in countries with hyper-endemic HIV such as South Africa, likely to foster increased HIV transmission through unprotected sex.

**Summary:**

The 2010 FIFA World Cup presents a strategic opportunity for South Africa to respond to the challenges that the sex industry poses in a strategic and rights-based manner. Public health goals and growing evidence on HIV prevention suggest that sex work is best approached in a context where it is decriminalised and where sex workers are empowered. In short, the authors argue for a moratorium on the enforcement of laws that persecute and victimise sex workers during the World Cup period.

## Background

Although a subject not usually broached by mainstream media or politicians, sex work has recently received increased attention in southern Africa. A Swaziland senator sparked public debate by suggesting sex work be legalised [1]. In Malawi, human rights non-governmental organisations (NGOs) are taking up a case against the police after they arrested 14 sex workers, forcibly tested them for HIV and reported their HIV results in the media [2]. The women were fined 1000 Malawian Kwatcha for trading in sex while having a sexually transmitted infection (STI). In the build-up to the FIFA 2010 World Cup in South Africa, alongside concerns about crime and the coaching of the South African football team, there has been consternation over an anticipated increase in demand for paid sex during the tournament [3,4]. Some have called for the temporary legalisation of sex work, while others have advocated a forceful crackdown on sex workers, involving mandatory HIV testing and sex worker registration with a regulatory authority [3-7].

Sex work is currently a criminal offence in most southern African countries [8] - as indeed it is in most of the world. Few health professionals have openly questioned whether criminalisation of sex work is a sound public health notion. These questions are particularly pertinent in southern Africa, a region with hyper-endemic HIV [9]. Rather than directly challenging legal frameworks, some health workers have sought to provide HIV prevention services for sex workers. This indirect approach has been encouraged by international funding agencies such as the US Presidential Emergency Plan for AIDS Relief (PEPFAR), which make funding conditional on a pledge by recipient organisations that they will not advocate for the legalisation of sex work [10-12]. Given the legal and funding impediments to the work of NGOs and the lack of government support for these initiatives, health care programmes have only managed scattered and broadly ineffective attempts at preventing HIV in sex workers in southern Africa, their clients and by extension, the general population [13,14].

## Discussion

### The laws of demand and supply

Sex work will not go away. A narrow market perspective suggests that demand for paid sex will be met by supply [15]. This may be especially true of settings with marked economic and gender inequities, as research by the International Labour Organisation indicates: "poverty has never prevented men from frequenting prostitutes, whose fees are geared to the purchasing power of their customers" [16]. Sociologists, economists and psychologists have argued for recognition of a number of factors that render the demand-supply approach to sex work more complex. These factors include: the social construction of sexuality; (female) bodies being available for (male) consumption; the existence of viable alternative employment opportunities for sex workers; the social stigma that attaches to sex work; and the role of global consumerism [17].

Sex work is not regarded as the oldest profession for nothing and demand will almost certainly grow with increased globalisation [6], regardless of the legal framework a country adopts. Among other things, demand is driven by the expansion of cultural conceptions of sex as a commodity, the increased movement of people and capital, and the rapid expansion of Information and Communication Technology [6].

### Sex worker vulnerability to HIV

Much of the vulnerability of sex workers to HIV in southern Africa stems directly from the criminalisation of their work and the patriarchal context in which they operate.

#### Limited access to services

Sex workers are often marginalised and face multiple barriers to accessing the health and social services they need: STI screening and treatment, HIV testing and tailored counselling, post-exposure prophylaxis after rape, access to male and female condoms, antiretroviral treatment, as well as mental health support and substance abuse treatment [18]. Health care workers with negative or prejudiced attitudes towards sex workers further restrict access to services [19-21] and drive sex workers away from treatment and support.

#### Sexual and gender-based violence

Sex workers commonly experience violence [22,23]. Criminalisation prevents sex workers from reporting abuse to the police or from seeking legal recourse after rape or sexual assault, which in turn serves to strengthen clients' power and dominance over them. Police harassment of sex workers is well-documented, and can take the form of assault, unlawful arrests, rape, extortion, and demands for sex or money as bribes [24-29].

#### Unsafe work conditions

The illegal nature of their work means that sex workers operate in risky and often crime-laden areas [30]. Such spaces are inherently dangerous, diminishing the likelihood that services and support structures for vulnerable populations will be established [31-33]. Their illegal position makes it near-impossible for sex workers to mobilise and form trade unions to effect collective change to their material conditions.

#### Difficulties with negotiating safer sex

Sex workers often report that it is difficult to persuade clients to use condoms and that they fear a violent reaction if they insist on condom use [24,34,35]. Some clients pay more for sex without a condom [24] or threaten to hire other sex workers who do not expect condom use [36]. With intense competition for clients weakening the bargaining power of individual workers, these factors all too often culminate in unsafe sex.

#### Stigma

Sex work is highly stigmatised in southern Africa - as it is elsewhere - and discrimination, violence and abuse against sex workers are often publicly condoned [28]. Health care and HIV programmes that focus solely on sex workers and overlook the role of clients, reinforce stigmatising discourses that sex workers are "vectors of the epidemic" and that safer sex should be the sole responsibility of sex workers themselves [37,38].

Not surprisingly, in the current stigmatising milieu of criminalisation, health interventions have had a limited effect on the lives of sex workers and, indeed, on the HIV epidemic. In contrast to the successful use of legal mechanisms to control public health hazards like tobacco, for example, few attempts have been made to use similar tactics to mitigate the HIV epidemic where it relates to sex work. This is surely a missed opportunity: history tells us that sensibly applied, legislative processes can be a most powerful public health ally. Equally, harmful laws may obstruct and hinder public health. From the onset of the HIV epidemic, wherever marginalised groups have been criminalized and stigmatised, be they men who have sex with men, intravenous drug users or people with HIV, these groups have been driven underground, away from essential health and social services, and towards HIV risk [19,39]. The same holds true for sex work, as illustrated by Table 1, which details the potential effects of decriminalisation on sex worker vulnerabilities, taking into account the current context in South Africa. The table summarises our main argument, namely, that where the control of criminal law and sexual moralism over sex work diminishes, so the reach of health, social and legal services to this population expands and positive public health benefits follow.

**Table 1:** Vulnerabilities of sex workers due to criminalisation: a situation analysis of South Africa and potential effects of decriminalisation

Vulnerabilities	Current situation in South Africa	Potential effects of decriminalisation and provision of public-sector services for HIV prevention in sex work settings
Restricted access to health services	Lack of specialized services targeting sex workers	Formal sex worker clinics and outreach, with active follow-up services
	Scanty and ineffective public and donor funding for HIV prevention in sex work settings	Public funding for HIV prevention in sex work settings, and government-led coordination of services
	Condom availability in general primary health clinics, but limited promotion of condoms in sex work settings	Targeted condom promotion and provision in sex work settings
	Syndromic treatment of symptoms within general STI services	Targeted STI control programmes with STI screening at pre-specified intervals, periodic presumptive treatment and syndromic management
	Limited access to health information and family planning counselling. High rates of unintended pregnancy, increasing number of dependents	Planned health promotion activities, with information provision, family planning counselling and contraceptive services
Restricted access to legal protection	Laws against gender-based violence are seldom enforced and police do not act on sex worker complaints	Sex workers have legal recourse to redress violence Enhanced ability of police to improve the safety of sex work settings
Unsafe work conditions	Unsafe venues	Enhanced ability to secure and control sex work settings
	Obtaining clients and negotiation often occurs in alcohol settings	Alcohol and paid sex can be delinked
	Difficulties in negotiating safe sex	More empowered sex workers enables condom negotiation and client refusal
Stigma	Judgemental health care workers	Specialized health care workers, trained in sensitive provision of services
Economic Vulnerabilities	Despite the threat of fines or imprisonment, women enter sex work in response to demand for paid sex and pressures of providing for dependents, as they have few alternatives	No evidence that decriminalisation will increase supply of sex workers or demand for sex work

### International sporting events and sex work

International sporting events are increasing in frequency and magnitude. It is estimated that the FIFA World Cup will bring 450 000 visitors to South Africa in 2010 [40] - the country with the highest number of people with HIV in the world [9]. Surprisingly little research has been conducted into the demand and supply of paid sex during big sporting events [41], and where the topic has been explored, the focus tends to fall on human trafficking for the purposes of sexual exploitation rather than on adult, consensual sex work. A recent report setting out recommendations for the 2010 Winter Olympics to be held in Vancouver, Canada, reviewed the available data and found that " [t]he commonly held notion of a link between mega sports events, TIP (Trafficking in Persons) and sex work is an unsubstantiated assumption."[41]. As evidenced by the media hype over trafficking in Germany during the 2006 World Cup [41,42], however, the sensationalism associated with human trafficking often dwarfs the more mundane, everyday concerns of consensual, adult sex work - demand for which tends to increase in host countries during big sporting events.

While there appears to be little evidence of increased trafficking during big sporting events, it is anticipated in South Africa that a number of tourists will combine soccer and tourist attractions with paid sex. We should not be surprised, therefore, when countries begin to warn their citizens travelling for the World Cup that sex in South Africa equates HIV. The press in England has already done so [5,43], while the Netherlands' State Secretary for Health, Welfare and Sport has warned Dutch football fans to bring their own condoms to South Africa as there may be a shortage during the World Cup [44]. Reducing transmission of HIV during this period, and the concerns of tourists and foreign governments around this, should thus be a priority for the tournament organisers. Our view is that reducing HIV transmission during this period would be best achieved in an environment where sex work is decriminalised. A pragmatic and human rights-based approach drawing on sound public health principles - not criminal and punitive sanction - is appropriate and timely.

### Decriminalisation and the 2010 FIFA World Cup

**Figure 1:**
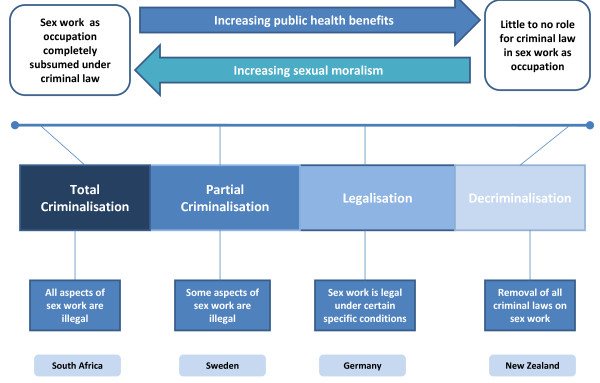
**Sex work and the role of criminal law**. As the role of criminal law diminishes in the control of sex work, so the public health benefits increase.

Many international bodies have already recognised the value of decriminalisation[45,46]. This approach has been supported by policy makers, legislators and scientific researchers alike [21,47,48]. Countries like Senegal, the Netherlands, Belgium, Australia and New Zealand have moved away from total criminalisation of sex work (see Figure 1). Yet, only New Zealand has explicitly decriminalised sex work, choosing instead to adopt a human rights and public health framework. The New Zealand Prostitution Reform Act was passed in 2003 and the effects of legislative change measured five years later. Contrary to public fears, no increase was found in the number of people entering sex work during this period [49,50]. Sex workers reported improved working conditions and wellbeing, feeling safer under the new legal framework, and being able to negotiate safer sex and report abuse to police [49].

Importantly, South Africa's *HIV & AIDS and STI Strategic Plan (2007-2011) *[51] recognises that several higher-risk groups, such as sex workers and drug users, face barriers to accessing HIV prevention and treatment services, and explicitly recommends the decriminalisation of sex work. And for almost a decade, the country has been reviewing its sexual offences legislation and considering reform of sex work laws [52,53]. It would have been prudent for these processes to have been concluded before the 2010 World Cup, but the South African Law Reform Commission will reportedly only release its recommendations on law reform and sex work in 2011 [54]. South Africa has missed an important opportunity: Germany, by contrast, proactively reformed its laws on sex work in 2002 - four years before hosting the FIFA World Cup.

Sporting events provide an opportunity to create a long-term legacy. In South Africa, for example, this will be improved road infrastructure and public transport. The groundswell of South African support for the World Cup likely has made the public more accepting of social change and the considerable short-term inconvenience caused by these legacy projects. It is still not too late to utilise the political capital afforded by the World Cup to galvanise policy processes that will approach sex work pragmatically and place public health benefits above ideological interests.

Given the limited time before kick-off, in lieu of decriminalisation we recommend a Parliamentary-sanctioned moratorium on the enforcement of laws that persecute and victimise sex workers during the World Cup period. This should be accompanied by initiatives that empower and support sex workers to insist on safer sex, such as the formation of collectives to assist sex workers in protecting themselves and their clients. Strong public health messages on safer sex, directed at the general population and incoming tourists in particular, should underlie these initiatives. This strategy would draw on lessons learnt during the Germany FIFA World Cup in 2006: distribution of free male and female condoms, and raising awareness on safer sex and sex worker rights during World Cup games are critical [55]. A moratorium on enforcing sex work laws and an implementation of sex work-specific programmes during the World Cup would provide important lessons that could inform long-term legal strategies around sex work, HIV and human rights in South Africa.

Following the World Cup, pressure should be increased on the South African Law Reform Commission to recommend full decriminalisation of sex work in South African law. These recommendations were echoed at a recent intersectoral consultation on sex work and the FIFA World Cup, held in Cape Town and attended by government, sex worker organisations, human rights advocates and public health researchers, during which various approaches to the World Cup were considered and a subsequent plan of action drafted [56].

## Conclusion

Decriminalising sex work is at odds with the sensibilities of many political and religious leaders and often raises their indignation and ire. Yet watching a population being decimated by HIV should evoke similar responses and elicit strong action based on evidence. We hope the post-Mbeki administration will attribute more weight to public health goods than an ideology based on sexual moralism - an ideology which, time and again, has been proven ineffective in preventing HIV in South Africa and beyond. Public discourses lamenting the "immorality" of sex work should be substituted for action that prioritises public health measures and legal frameworks which secure the long-term health of South Africans.

In conclusion, the FIFA World Cup presents a strategic opportunity for South Africa to respond to challenges posed by the sex industry in a strategic and rights-based way. Public health goals and available evidence suggest that sex work is best approached in a context where it is decriminalised and where sex workers are empowered, not victimised or persecuted (Table 1). Attention to improving sex worker access to the health and social services they need to prevent infection will do more to prevent HIV transmission than misguided attempts to legislate sex work out of existence. A sensible South African response to sex work in the context of a global celebration of soccer could inspire long-term progressive changes to its legal framework and encourage the rest of southern Africa to follow suit.

## Competing interests

The authors declare that they have no competing interests.

## Author information

Fiona Scorgie and Richard Steen are independent consultants.

## Authors' contributions

MR presented some of the initial ideas on legal frameworks and sex work at an Inter-Agency Consultation on HIV prevention in the context of Sex Work, hosted by the United Nations Population Fund (5-7 October 2009, Esibayeni lodge, Manzini, Swaziland). MC conceived of this article and engaged MR, FS, SL, RS and MT in dialogue and correspondence on the ideas. MR outlined the article in point format and MC and FS contributed feedback and substantive intellectual input in developing the argument. MR and MC then drafted the manuscript and received input from FS, SL, RS and MT. All authors edited and proofread the final manuscript.
